# Supplementary figures and images for: A high-quality severe combined immunodeficiency (SCID) rat bioresource
Source: PLoS One. 2022 Aug 12;17(8):e0272950. doi: 10.1371/journal.pone.0272950 (PMC9374221; doi:10.1371/journal.pone.0272950)

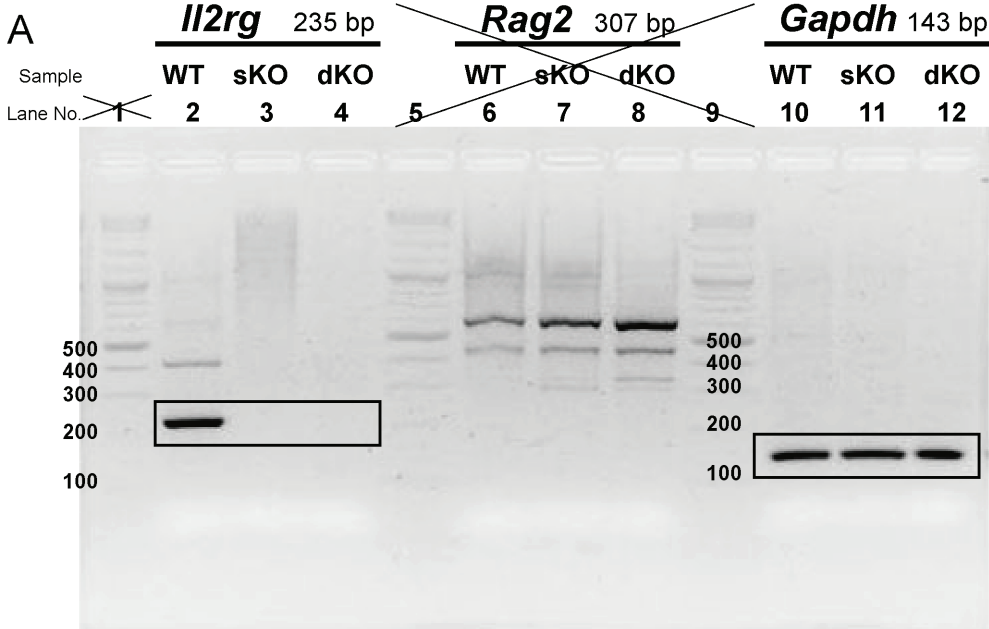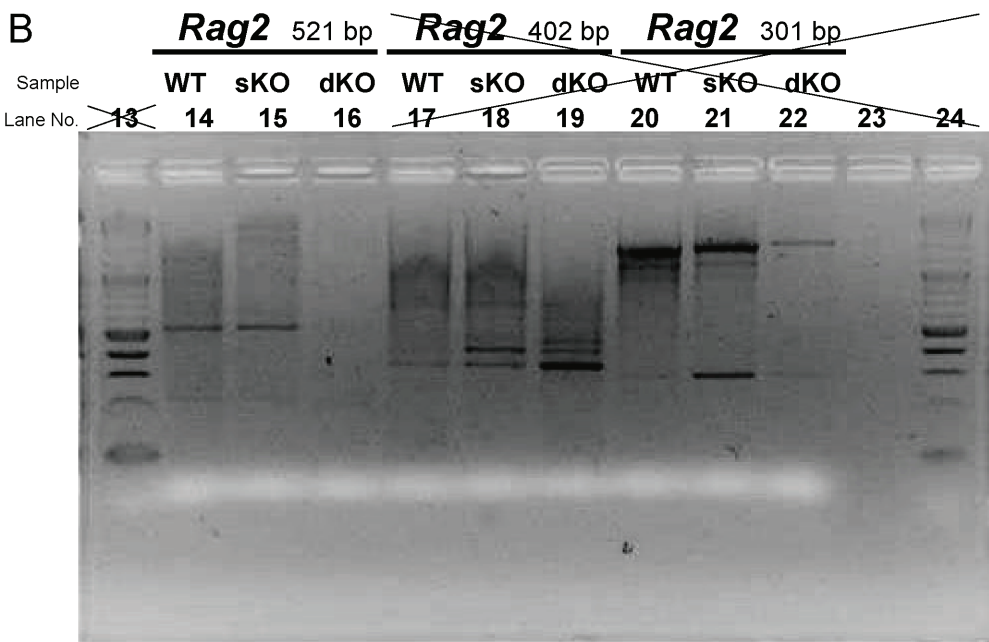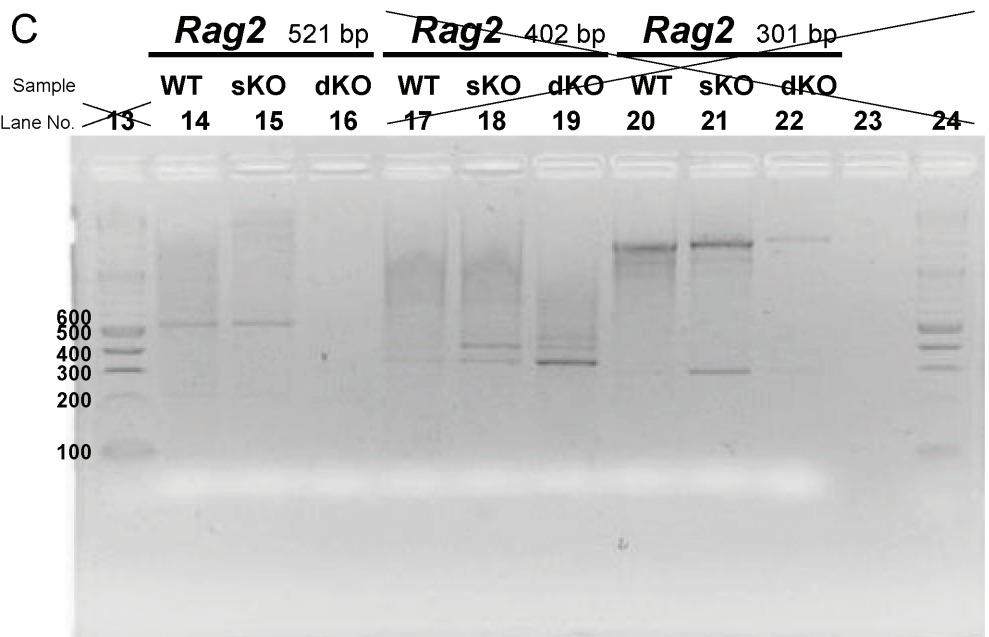

Supplement: S1 Fig — Three animals per group were randomly selected for RT-PCR analysis. DNA samples were separated by electrophoresis in 1.5% agarose gel; the gels were stained with Invitrogen™ SYBR™ Safe DNA Gel Stain (Thermo Fisher), imaged under the blue/green LED light (Blue/Green LED transilluminator, Nippon Genetics CO., LTD. Tokyo, Japan) by a gel imager (Gel Scene GST-33, Astec CO., Ltd. Fukuoka, Japan). Image A was analyzed without adjustment. Image B (used for analysis) is an automatic contrast-adjusted version of image C; image C is the unaltered version of image B. Lanes 1, 5, 9, 13 and 24 contain DNA size markers. Lane No. 23 is a blank lane. Rectangular outline areas in images A and B were cropped (Il2rg: Lanes 2, 3, 4; Gapdh: Lanes 10, 11, 12 in image A. Rag2: Lanes 14, 15, 16 in image B) and combined to form Fig 1C. Rag2 electrophoresis was performed using distinct primers for image A vs. images B and C. Lanes 14, 15, and 16, which demonstrated specific amplification, are shown in Fig 1C. (PDF) [file pone.0272950.s001.pdf]
