# Supplementary material for: A high-quality severe combined immunodeficiency (SCID) rat bioresource
Source: PLoS One. 2022 Aug 12;17(8):e0272950. doi: 10.1371/journal.pone.0272950 (PMC9374221; doi:10.1371/journal.pone.0272950)
Supplement: Attachment — Submitted filename: comment PONE-D-21-27266.docx [file pone.0272950.s002.docx]

Comment

The manuscript titled “Development of a high-quality bio-resource of severe combined immunodeficient (SCID) rat models with Il2rg and/or Rag2 mutations” by Yoshiki Miyasaka et al, aimed to describe the generation of severe combined immunodeficient (SCID) rat models by knockout of the interleukin-2 receptor gamma chain gene (Il2rg) independently or together with the recombination activating gene 2 (Rag2) using the CRISPR/Cas9 genome editing system. And the generated SCID rats was confirmed by multiple methods.

This study successfully established a severe immunodeficiency rat model. This model showed significantly reduced immune cells without significant growth retardation or defective gametogenesis. It is undoubtedly of positive significance for humanized studies. Comments are the following:

1. Line 164 Page 11, for the generation of SCID rat, it is better to present a schematic diagram of workflow.
2. Line 155 Page 8, The list of tumors seems redundant. It is more appropriate to show it in the table. In addition, the abbreviation for the tumor name is repeated in the figure legend (Figure 2 & 3).
3. In Figure 5, left panel and right panel need to be labeled with gender information.
4. Please label the statistical comparison results in figures (Fig. 2c and Figure 5) and indicate the statistical method used in the legend.
5. The writing and the resolution of the figures in this manuscript could to be improved.
